# *Notes from the Field: Trichophyton mentagrophytes* Genotype VII — New York City, April–July 2024

**DOI:** 10.15585/mmwr.mm7343a5

**Published:** 2024-10-31

**Authors:** Jason Zucker, Avrom S. Caplan, Shauna H. Gunaratne, Stephanie M. Gallitano, John G. Zampella, Caitlin Otto, Rachel Sally, Sudha Chaturvedi, Brittany O’Brien, Gabrielle C. Todd, Priyanka Anand, Laura A.S. Quilter, Dallas J. Smith, Tom Chiller, Shawn R. Lockhart, Meghan Lyman, Preeti Pathela, Jeremy A.W. Gold

**Affiliations:** ^1^Division of Infectious Diseases, Columbia University Irving Medical Center, New York, New York; ^2^The Ronald O. Perelman Department of Dermatology, NYU Grossman School of Medicine, New York, New York; ^3^Department of Dermatology, Columbia University Irving Medical Center, New York, New York; ^4^Department of Pathology, NYU Grossman School of Medicine, New York, New York; ^5^Wadsworth Center, New York State Department of Health; ^6^Department of Biomedical Sciences, School of Public Health, University at Albany, Albany, New York; ^7^Division of STD Prevention, National Center for HIV, Viral Hepatitis, STD, and TB Prevention, CDC; ^8^Epidemic Intelligence Service, CDC; ^9^Division of Foodborne, Waterborne, and Environmental Diseases, National Center for Emerging and Zoonotic Infectious Diseases, CDC; ^10^New York City Department of Health and Mental Hygiene, New York, New York.

**Summary**

**What is already known about this topic?**

*Trichophyton mentagrophytes* genotype VII (TMVII), an emerging fungus, causes genital tinea that can be spread through sex and might require prolonged treatment. The first U.S. case was reported in June 2024.

**What is added by this report?**

Four additional TMVII infections were diagnosed during April–July 2024 in New York City among men who have sex with men. Tinea occurred on the face, buttocks, or genitals and was successfully treated with antifungal medications.

**What are the implications for public health practice?**

Health care providers should be aware that TMVII is an emerging infection spread through sex.

*Trichophyton mentagrophytes* genotype VII (TMVII) is an emerging dermatophyte fungus, causing tinea that can be spread through sexual contact (*1*). TMVII can cause pruritic, annular, scaly lesions on the trunk, groin, genitals, or face; might be mistaken for eczema, psoriasis, or other dermatologic conditions; and frequently requires oral antifungal therapy.^†^ Some patients experience inflamed, painful, and persistent lesions that can lead to scarring or secondary bacterial infection. TMVII infections have been reported among men who have sex with men in France since March 2021 and previously in men who traveled to Southeast Asia for sex tourism (*1*,*2*). In June 2024, a TMVII case in the United States was reported in a man who developed genital lesions after traveling to several countries in Europe and to California and who had sexual contact with multiple men while traveling (*3*). Clinicians subsequently alerted public health officials of additional patients in the United States who had laboratory-confirmed TMVII infection.

## Investigation and Outcomes

During April–July 2024, four additional patients received a clinical diagnosis of tinea. Samples were collected for fungal culture; following growth, TMVII was identified using Sanger sequencing of the internal transcribed spacer region of the ribosomal gene. Antifungal susceptibility testing was performed using previously described methods (*3*). For each patient’s isolate, the minimum inhibitory concentrations of terbinafine and itraconazole were 0.0039 mg/mL and <0.03 mg/mL, respectively, suggesting susceptibility to these drugs. Patients were treated for presumed TMVII infection before laboratory confirmation and antifungal susceptibility results were available. Data on demographic characteristics, potential exposures, clinical course, and current infection status were obtained as part of clinical care. This activity was reviewed by CDC, deemed not research, and was conducted consistent with applicable federal law and CDC policy.^§^

All four patients were cisgender men aged 30–39 years who reported recent sexual contact with other men. Patients A and D reported sexual contact with each other; patients B and C had no known epidemiologic link to anyone with known TMVII infection. Patient D was a sex worker. Patient B reported travel to Europe; the other patients reported no recent international travel history. Each patient was screened for other concomitant sexually transmitted infections and received negative test results.

Patient A had no underlying medical conditions and was taking HIV preexposure prophylaxis. He was initially evaluated for a rash on his buttocks. He completed 2 weeks of topical clotrimazole followed by 1 week of topical terbinafine with no improvement. He then was prescribed oral terbinafine (250 mg daily) for a planned 2–4-week course. At last follow-up, the rash was improving.

Patient B had HIV infection and had inconsistent adherence to antiretroviral therapy. He was initially evaluated for a pruritic rash on the corner of his mouth. He completed 1 week of topical clotrimazole with complete resolution of his rash.

Patient C had HIV infection that was well-controlled on antiretroviral medication. He was initially evaluated for a rash on his knee, buttocks, and groin and was prescribed oral terbinafine (250 mg daily) with a planned 4-week course. The rash was improving at last follow-up.

Patient D was taking HIV preexposure prophylaxis and dabrafenib/trametinib for a history of cancer. He was initially evaluated for a pruritic rash on his knee, trunk, arm, and penile shaft (Figure). He completed <1 week of oral terbinafine (250 mg daily) and was switched to itraconazole (200 mg twice daily), topical luliconazole, and topical ketoconazole. The rash was improving at last follow-up.

**FIGURE:**
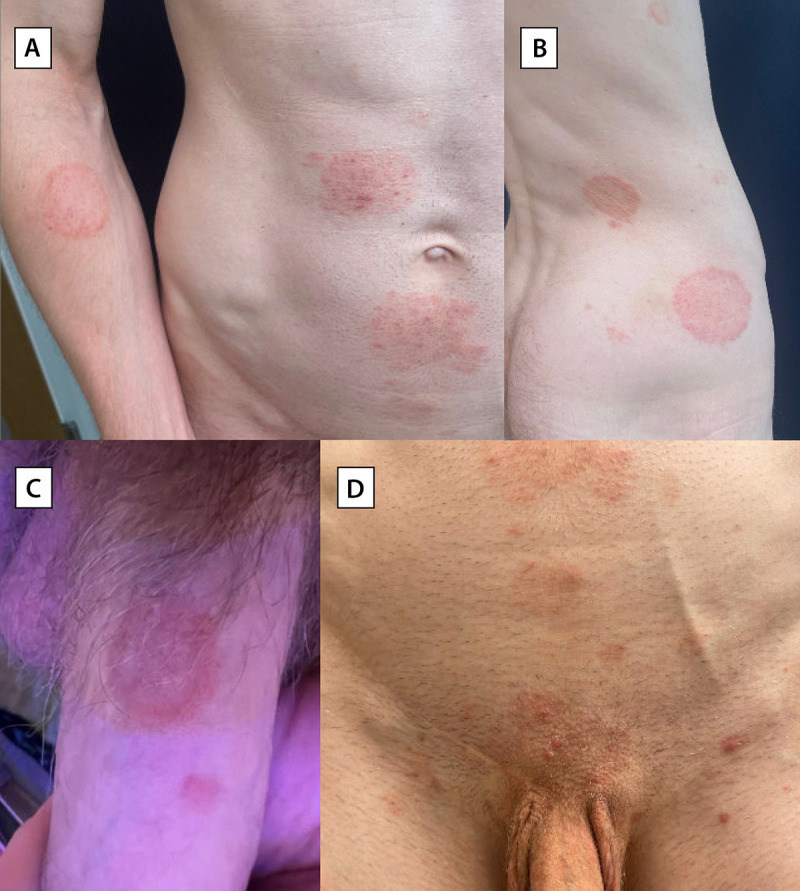
Dermatology evaluation of a patient with *Trichophyton mentagrophytes* genotype VII rash, showing tinea corporis* (A and B), tinea genitalis**^†^** (C), and tinea corporis and tinea pubogenitalis (D)**^§^** — New York City, April–July 2024 Photos/Avrom S. Caplan (used with patient’s permission) * Annular, scaly plaques on the right arm and trunk. ^†^ Annular, scaly plaque on the penis. ^§^ Scaly plaques on trunk and genital area, with scattered erythematous papulonodules likely indicating follicular involvement of dermatophytosis.

## Preliminary Conclusions and Actions

Health care providers should be aware that TMVII can spread through sexual contact and cause lesions on the genitals, buttocks, face, trunk, or extremities.^¶^^,^** Point-of-care testing with direct microscopy can help confirm tinea (*4*); however, TMVII identification requires fungal culture and DNA sequencing. Clinicians should initiate empiric therapy based on epidemiologic and clinical features, especially given the insensitivity of fungal cultures (*5*) and that confirmation of TMVII infection might take weeks. Current evidence suggests that oral terbinafine (250 mg daily) is an effective first-line option for TMVII infections. Some patients have also been successfully treated with itraconazole with adjuvant topical antifungal therapy. Patients might require oral antifungal therapy for up to 3 months and should take the treatment until lesions have fully resolved (*1*,*3*). Some patients might respond to topical antifungals alone, but these are not recommended as monotherapy for tinea involving hair follicles (*4*).

Health care providers should advise patients with TMVII infection about the importance of avoiding skin-to-skin contact with affected areas and not sharing personal items until symptom resolution. Topical corticosteroid products, including combination antifungal-corticosteroid products, can worsen tinea infection and should be avoided in treating TMVII and other dermatophyte infections (*4*). Public health surveillance, health care provider and patient education and awareness, and increased access to dermatophyte identification and antifungal susceptibility testing could help detect, monitor, and prevent the spread of TMVII.

## Footnotes

* These authors contributed equally to this report.

† Typically, dermatophyte infections of the skin, also called tinea or ringworm, are mild conditions that can be treated with topical antifungals and do not affect the genitals. TMVII might have a predilection for anogenital skin. TMVII is distinct from *Trichophyton indotineae*, another emerging dermatophyte fungus that can also cause severe tinea but 1) affects the genitals less frequently; 2) is generally resistant to terbinafine, the first-line therapy for TMVII infection; and 3) in the United States, is usually associated with travel and immigration involving Southeast Asia. https://www.cdc.gov/ringworm/aboutemergingringworm/index.html

§ 45 C.F.R. part 46.102(l)(2), 21 C.F.R. part 56; 42 U.S.C. Sect. 241(d); 5 U.S.C. Sect. 552a; 44 U.S.C. Sect. 3501 et seq.

¶ Health care providers who suspect TMVII infection can contact their state and local health departments for assistance (https://www.cdc.gov/public-health-gateway/php/index.html) and can request consultation for the evaluation and management of challenging sexually transmitted infection cases from the National Network of STD Clinical Prevention Training Centers STD Clinical Consultation Network, which is supported by CDC’s Division of STD Prevention (https://www.stdccn.org/render/Public). Public health officials concerned about potential cases of antifungal-resistant ringworm or unusual clusters of cases can email FungalOutbreaks@cdc.gov for assistance.

** Information about TMVII infection recognition, diagnosis, and treatment is available online (https://www.aad.org/member/clinical-quality/clinical-care/emerging-diseases/dermatophytes/other-emerging-dermatophytes), including information on laboratories that perform confirmatory testing (https://www.aad.org/member/clinical-quality/clinical-care/emerging-diseases/dermatophytes/recognizing-trichophyton-indotineae). Information on these websites was developed as a collaboration between CDC and The American Academy of Dermatology Emerging Diseases Task Force (https://www.aad.org/member/clinical-quality/clinical-care/emerging-diseases).
